# Strengthening the Quality and Quantity of the Nursing and Midwifery Workforce: Report on Eight Years of the NEPI Project

**DOI:** 10.29024/aogh.6

**Published:** 2018-04-30

**Authors:** Susan Michaels-Strasser, Janel Smith, Judy Khanyola, Roberta Sutton, Tashtiana Price, Wafaa M. El Sadr

**Affiliations:** 1 ICAP at Columbia University, New York, US; 2 Health Resources and Services Administration (HRSA), US

## Abstract

In response to the urgent need to scale up access to antiretroviral therapy, the Global Nursing Education Partnership Initiative (GNCBP), a PEPFAR program administered by the U.S. Department of Health Resources and Services Administration (HRSA), was implemented from 2011 to 2018 by ICAP at Columbia University. Working closely together, HRSA and ICAP partnered with local nursing leaders and ministries of health to strengthen the nursing and midwifery workforce across 11 countries. This multi-country project, developed to address critical gaps in nursing education and training worked across six building blocks of health workforce strengthening: infrastructure improvement, curricula revision, clinical skills development, in-service training, faculty development and building partnerships for policy and regulation to increase the quality and quantity of the nursing and midwifery workforce. As a result, 13,387 nursing and midwifery students graduated from schools supported under GNCBP. A total of 5,554 nurses received critical in-service training and 4,886 faculty, clinical mentors and preceptors received training in key clinical care areas and modern teaching methodologies. ICAP completed 43 infrastructure enhancements to ensure environments conducive to learning and strengthened nursing leaders as best evidenced by the election and formation of Mozambique’s first national nursing council and the NEPI Network. Going forward, efforts to strengthen nursing and midwifery can build on the results of the GNCBP project. Going forward, a new group of African nursing leaders are being supported to advocate for high quality patient-care led through inter-professional collaboration and participation in international efforts championing the critical role of nurses in achieving universal health coverage.

## Introduction

To achieve the UNAIDS [1] 90-90-90 goals, whereby 90% of HIV positive people know their status, 90% of those diagnosed are on treatment and 90% of those on treatment are virally suppressed, will require well-run health facilities and a skilled workforce supported by a strong health system. As nurses provide the majority of primary health services, global goals to achieve HIV epidemic control will require a highly skilled and well-equipped nursing workforce [2]. Yet critical gaps in HIV nursing education and training have been identified [3]. The gaps in both the quality and the quantity of the global health workforce education led the United States Congress to enact legislation in 2008 which has included a commitment to train and retain at least 140,000 new health care professionals and paraprofessionals emphasizing the training and deployment of doctors and nurses to deliver primary health care [4].

## Background

**Figure 1:**
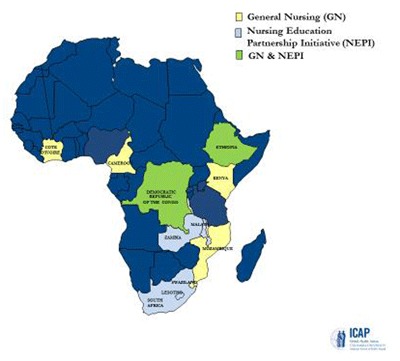
Countries engaged in the Global Nurse Capacity Building Project.

In response, the Global Nursing Education Partnership Initiative (GNCBP), a PEPFAR program administered by the U.S. Department of Health Resources and Services Administration (HRSA), was implemented by ICAP at Columbia University. Working closely together, HRSA and ICAP have partnered with local leaders to strengthen the nursing and midwifery workforce. This multi-country project, developed to address critical gaps in nursing education and training, was launched in 2011, with the field work completed in September 2017. The GNCBP spanned 11 countries and included two subprojects; the Nursing Education Partnership Initiative (NEPI) and General Nursing (GN). The GNCBP supported local projects, institutions, and networks to expand, enhance, and sustain the nursing and midwifery workforce through NEPI and GN (Figure 1).

This paper presents the approach and overall results of GNCBP. Special attention is given to the NEPI sub-project; a counterpart initiative to the Medical Education Partnership Initiative (MEPI). The purpose of NEPI was to strengthen both the quality and quantity of the nursing and midwifery workforce through strengthened nursing and midwifery education. The NEPI project had five broad objectives: 1) strengthen teaching/learning infrastructure; 2) improve quality and relevance of teaching and learning; 3) improve the capacity of nursing and midwifery faculty; 4) build governance, leadership and administrative capacity within education institutions; and 5) enhance partnerships and networks. With the release in 2014 of PEPFAR 3.0 – *Controlling the Epidemic: Delivering on the Promise of an AIDS-free Generation*, commonly referred to as the PEPFAR pivot, increased attention was given to HIV specific education interventions for students and faculty, while continuing to ensure that the basic building blocks of nursing education and training were in place at NEPI-supported schools [5]. Reducing the theory-to-practice gap in nursing and midwifery and increasing the number of frontline health workers to meet population health priorities was recognized as critical to both achieving antiretroviral treatment scale-up and 90-90-90.

## Methodology

**Figure 2:**
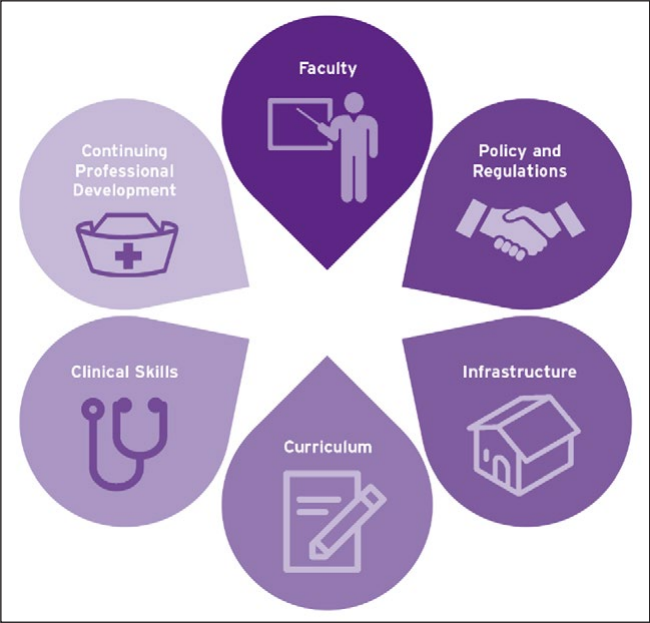
ICAP’s Six Building Blocks for Strengthened Nursing Education and Training.

Across the six countries supported by NEPI, a total of twenty-two schools of nursing participated (Table 1). Schools were chosen through a consultative process led by local stakeholders. Interventions were prioritized by country specific NEPI technical working groups (TWGs) following external baseline needs assessments. In line with the outcome of a WHO and PEPFAR consultative meeting, NEPI TWG interventions focused on six areas of HRH capacity building: infrastructure improvement, curricula revision, clinical skills development, in-service training, faculty development and building partnerships for policy and regulation [6] (Figure 2). Each of the six building blocks was seen as fundamental to the scale up of quality nursing and midwifery education. The schools supported by NEPI received technical support from ICAP in program design, implementation, clinical training, monitoring, evaluation, research, administration, and grant management. Tuition support, where it was deemed vital to student enrollment and retention, was provided. With PEPFAR 3.0, a stronger focus on Nurse Initiated and Managed Antiretroviral Therapy (NIMART) competence was realized through NIMART training for educators and students, curriculum review and updates for PEPFAR priorities, such as key populations and adolescents. A series of three HIV specific e-learning programs have been developed as well as the development and use of an HIV core competency assessment tool.

**Table 1:** NEPI-Supported Nursing Education Institutions by Country, 2012–2017.

Country	Schools

DRC	Kinshasa Higher Institute of Medical Technology
	Lubumbashi ISTM
	Kintambo Technical Medical Institute
	Kamalondo Institute of Medical Technology
Ethiopia	University of Gondar
	University of Addis Ababa
	Arba Minch College of Health Sciences
Lesotho	National University of Lesotho
	National Health Training College
	Schools of Nursing in Maluti, Roma, Scott, Paray
Malawi	Mzuzu University, Department of Nursing and Midwifery
	University of Malawi, Kamuzu College of Nursing
	Malawi College of Health Sciences
South Africa	Free State School of Nursing, Northern Campus
	Mpumalanga College of Nursing
	Prince Mshiyeni Hospital College of Nursing
Zambia	University of Zambia, Department of Nursing Science
	Lusaka School of Nursing
	Monze School of Nursing

A brief description of nursing and midwifery strengthening efforts within each building block is provided below.

### Faculty

Faculty development focused on improving the ability of faculty to lead quality education programs and in later years to provide greater support and attention to nursing and midwifery education locally and regionally. Support was provided for a wide array of educational opportunities, including but not limited to, in-service training initiatives, such as nurse initiated and managed ART (NIMART), training in the set-up and use of simulation-based education and training, development of competency-based curricula, the nursing process, as well as tuition support for those nurses pursuing Master’s and PhD degrees. In the final year of NEPI, a leadership webinar series was undertaken, as well as an in-person nursing and midwifery leadership development workshop.

### Building Partnerships for Policy and Regulation

Robust nursing education and leadership networks were developed across NEPI-supported institutions and active links to regional and international nursing bodies have been formed through local technical working groups, the ‘NEPI network’, and through supporting local, regional and international conference attendance.

### Infrastructure

Prior to the NEPI program, nursing schools lacked the basic equipment and supplies including textbooks, journals and lecture halls able to accommodate an increased number of students. WHO, in the 2006 World Health Report, recognized the importance of increasing the size of the health workforce but at the same time improving educational infrastructure [7]. “Insufficiencies in infrastructure may place a significant constraint on the numbers of students who can be taught effectively and limit expansion of training, even for basic services [8].” Infrastructure needs were prioritized by the local NEPI technical working groups. Despite country led prioritization, nearly all countries and schools identified similar infrastructure needs, including housing for students, improved access to up-to-date textbooks, on-line journals, well-equipped lecture rooms and practical training rooms. Access to clinical practice sites, especially rural practice settings, was a specific concern.

### Curriculum

Nursing and midwifery curricula needed to be updated to reflect nurses and midwives current scope of practice and ‘real world demands’. We used the backward design to curriculum development was employed throughout the network of schools [9]. Educators and ICAP in-country staff were capacitated to use the backward design approach through a webinar lecture series and technical assistance. Emphasis was placed on the identification of competencies and learning objectives rooted in clinical practice demands. Incorporation of nursing competencies in line with increased task sharing focused on key HIV activities, such as HIV counseling and testing, prevention of mother to child transmission (PMTCT), care of the HIV exposed infant (HEI) and antiretroviral therapy. The development of competency-based curricula reflective of clinical practice was seen as fundamental to reducing the theory-practice gap.

### Clinical Skills

Within the NEPI project, ensuring that nursing students were both competent and confident was prioritized. Competency-based curricula were coupled with improved clinical skills training through both simulations and supervised clinical practice. The development of simulation-based education (SBE) included the procurement, set-up and training on use of SBE. Training was provided by both the equipment supplier, as well as regional faculty trained in SBE. High, medium and low fidelity equipment were acquired to ensure students had the ability to learn core skills in a controlled practice environment. Mannequins able to simulate routine nursing functions, such as venipuncture and intravenous infusion were complemented with higher fidelity mannequins that could simulate life-threatening conditions, such as emergency obstetric complications. Faculty were trained in the use of a wide range of equipment, including computer aided simulation which allowed dynamic manipulation of the ‘patients’ condition. Such features enable students to acquire experience and skills to be able to more competently deal with rare events, such as post-partum haemorrhage, as well as allowing faculty to assess student’s competence for real world challenges. In addition to SBE, NEPI country programs strengthened clinical training through preceptor education and training, improved access to rural practice settings, and allowed for the development and use of model wards.

### Continuing Professional Development

Continuing professional development (CPD) included increasing faculty access to HIV competency-based e-learning, with a pilot of Option B+ e-learning at NEPI supported schools in Lesotho, Zambia and Malawi and NIMART in-service training. Local, regional and international nursing conference participation and abstract submission was encouraged. Over the life of the project, NEPI attended and presented as such conferences as ECSACON (the East, Central and Southern Africa Colleges of Nursing), Sigma Theta Tau Honor Society of Nursing, the International Council of Nurses (ICN) and the Association of Nurses in AIDS Care (ANAC). With the PEPFAR Pivot, CPD courses and abstracts focused on HIV priority areas.

## Results

We present results across the six building blocks is presented here. (Table 2) The PEPFAR indicator for preservice education was tracked across all NEPI countries and schools [10]. The indicator refers to the number of new health workers graduating from a pre-service training program as a result of PEPFAR support [11]. Under GNCBP, a total of 13,387 nursing and midwifery students graduated from schools supported under the NEPI and GN sub-project. A total of 5,554 nurses received critical in-service training, and 4,886 faculty, clinical mentors and preceptors received training in key clinical care areas and modern teaching methodologies. ICAP completed 43 infrastructure enhancements to ensure environments conducive to learning, as well as the election and formation of Mozambique’s first national nursing council.

**Table 2:** Results across ICAP’s Six Building Blocks for Strengthened Nursing Education and Training.


***Faculty Development***	Faculty training included in-service training, training in curriculum reform and competency-based curriculum development, as well as continued education at the specialty certificate, Master’s and PhD levels.
***Building Partnerships for Policy & Regulation***	Over the life of the project a robust network was formed with active collaboration between beneficiaries and local partners. Partnerships were strengthened between nursing and midwifery leaders and stakeholders within Ministries of Health, Ministries of Education, Nursing and Midwifery Councils, and Nursing and Midwifery Professional Associations. Engagements were made with regional and international bodies, including but not limited to, the inter-professional body- AFREhealth, the Forum of University Deans in South Africa (FUNDISA), the East, Central and Southern Africa Colleges of Nursing (ECSACON), the International AIDS Society (IAS), the International Council of Nurses (ICN), the International Confederation of Midwives (ICM) and the Association of Nurses in AIDS Care (ANAC).
***Infrastructure***	A total of 43 infrastructure projects have been completed, including student hostels, computer labs, internet connectivity, clinical simulation laboratories and libraries.
***Curriculum***	Over 56 nationally accredited curricula were implemented, including a combined registered nurse-midwife program in Zambia, which removed duplication and reduced training from 6 to 3.5 years. Within South Africa, three new courses were developed for nursing college students. These courses are currently in review by the South Africa Nursing Council for degree level accreditation.
***Clinical Skills***	Clinical skills training was enhanced in NEPI-supported schools through e-learning to provide updates in new clinical guidelines, clinical simulation labs to enhance students’ clinical skills and confidence before entering the clinical setting, model wards with trained preceptors to provide students with quality clinical training and supervision in the health facility setting.
***CPD***	Three e-learning courses were developed to provide updates in key HIV areas, including Option B+ for prevention of mother to child transmission (PMTCT), Test and Start, and Pediatric Antiretroviral Therapy.


## Discussion

By supporting the education and training of over 13,000 new nurses and midwives, the NEPI program has made a significant contribution by increasing both the quality and the quantity of the health-care workforce in sub-Saharan Africa and strengthening the health care workforce’s ability to respond to HIV [12]. In addition to increasing the quantity of nurses and midwives produced, NEPI enhanced the quality of education through a standardized and comprehensive approach – developing six key building blocks of education, including infrastructure, curriculum, faculty, clinical skills, continuing professional development, and professional partnerships for policy and regulation. NEPI and GN interventions were increasingly tailored to address HIV in response to the 2014 PEPFAR pivot. ICAP, through country-led TWGs, engaged local stakeholders to ensure interventions were country-led and country-owned. Lessons learned from the NEPI program provide critical insight into optimal strategies and methods for infrastructure enhancement, faculty development, curriculum reform, clinical skills training, continuing professional development, and strengthened policy and regulation. Despite increasing the quality and the quantity of the nursing and midwifery workforce, further investment is needed to build on NEPI’s work to enhance nursing and midwifery education. Many schools of nursing and midwifery still lack the basic building blocks needed for quality nursing education. The lack of basic educational resources is in stark contrast to the critical front line role nurses and midwives play towards achieving epidemic control. Going forward, efforts to strengthen nursing and midwifery can build on the results of the GNCBP project including continued efforts to support the six building blocks for workforce strengthening. The eight-year GNCBP has supported a new cadre of nursing and midwifery leaders across Africa who are coming together as advocates for high-quality patient care led by nurses and midwives at the front lines of service delivery. Increasingly these leaders are engaging across professions as seen with the emergence of AfreHealth, a new inter-professional effort [13] and across continents through the *Nursing Now* Campaign, which builds on the Triple Impact report championing the critical role of nurses in achieving universal health coverage [14].

## Conclusion

Nurses provide the majority of HIV services in Africa. Epidemic control will not be achieved without a strong nursing and midwifery workforce at the front lines of primary health care. The NEPI project is the first large-scale systematic multi-country investment in Africa to strengthen nursing and midwifery education reflective of clinical practice. NEPI leaders now drive education and practice change at 22 schools of nursing in Africa, equipped with the basic building blocks for quality education. Recent inter-professional collaboration efforts are building partnerships across professions to drive improvement in education, research and service delivery. Yet, much work remains to ensure nursing and midwifery strengthening is sustained and shared with the thousands of nursing and midwifery schools that lack the basic building blocks of high quality education. The critical role of nurses in HIV must continue to be met with substantive investment in nursing and midwifery education.
